# Flow Front Monitoring in High-Pressure Resin Transfer Molding Using Phased Array Ultrasonic Testing to Optimize Mold Filling Simulations

**DOI:** 10.3390/ma17010207

**Published:** 2023-12-30

**Authors:** Linus Littner, Richard Protz, Eckart Kunze, Yannick Bernhardt, Marc Kreutzbruck, Maik Gude

**Affiliations:** 1 Institut für Kunststofftechnik (IKT), University of Stuttgart, 70569 Stuttgart, Germany; 2 Institute of Lightweight Engineering and Polymer Technology (ILK), Dresden University of Technology, 01307 Dresden, Germany

**Keywords:** thick-walled composite, ultrasonic testing, process optimization, HP-RTM, inline measurement, flow front monitoring, flow simulation

## Abstract

During the production of fiber-reinforced plastics using resin transfer molding (RTM), various characteristic defects and flaws can occur, such as fiber displacement and fiber waviness. Particularly in high-pressure RTM (HP-RTM), fiber misalignments are generated during infiltration by local peaks in the flow rate, leading to a significant reduction in the mechanical properties. To minimize or avoid this effect, the manufacturing process must be well controlled. Simulative approaches allow for a basic design of the mold filling process; however, due to the high number of influencing variables, the real behavior cannot be exactly reproduced. The focus of this work is on flow front monitoring in an HP-RTM mold using phased array ultrasonic testing. By using an established non-destructive testing instrument, the effort required for integration into the manufacturing process can be significantly reduced. For this purpose, investigations were carried out during the production of test specimens composed of glass fiber-reinforced polyurethane resin. Specifically, a phased array ultrasonic probe was used to record individual line scans over the form filling time. Taking into account the specifications of the probe used in these experiments, an area of 48.45 mm was inspected with a spatial resolution of 0.85 mm derived from the pitch. Due to the aperture that had to be applied to improve the signal-to-noise ratio, an averaging of the measured values similar to a moving average over a window of 6.8 mm had to be considered. By varying the orientation of the phased array probe and therefore the orientation of the line scans, it is possible to determine the local flow velocities of the matrix system during mold filling. Furthermore, process simulation studies with locally varying fiber volume contents were carried out. Despite the locally limited measuring range of the monitoring method presented, conclusions about the global flow behavior in a large mold can be drawn by comparing the experimentally determined results with the process simulation studies. The agreement between the measurement and simulation was thus improved by around 70%.

## 1. Introduction

Fiber-reinforced composites (FRPs) have been among the high-performance materials in the field of lightweight construction for several decades. Their mass-specific properties are the main reason for their use in fields such as aerospace, wind energy, trains, and automotives. The mass-specific strength or modulus of elasticity are significantly higher than most metallic alloys, allowing for unparalleled efficiency. The properties of FRPs are strongly influenced by the fibers, matrix material, and laminate structure, among other factors [1]. There are a variety of molding processes used to produce FRPs depending on part geometry and quantity. Typical processes include hand lay-up processing, filament winding, pultrusion, autoclave processing, and resin transfer molding (RTM). RTM is a resin injection process that uses mainly low-viscosity thermoset resin systems such as epoxy resins or polyurethanes. To reinforce the components, a near-net-shape fiber semi-finished product, also known as a preform, is clamped in a closed mold. The resin system is preheated and premixed in a mixing chamber and then injected into the mold with pressure (1–20 bar). In addition, a vacuum is applied to increase the diffusion of the resin. As a result, the incoming matrix system completely encloses the previously dry fiber structure and subsequently cures. Out of the processes already mentioned, RTM has the greatest potential to meet the requirements for large production volumes, as demanded in the automotive and aviation sectors. This is mainly due to the fact that RTM can be used to produce three-dimensional, complex, and large composite parts with low tolerances and a high degree of automation.

High-pressure resin transfer molding (HP-RTM) is considered to be a further development of RTM. By increasing the injection pressure up to 200 bar and through the resulting increase in the flow rates of the resin system, HP-RTM enables the use of fast-curing duromer-based resins, resulting in a drastic reduction in cycle time. However, the high flow rates lead to an increase in the probability of defects in the form of fiber disorientation and fiber waviness due to local flow rate peaks, which increase again as the wall thickness of the FRP increases too [2]. To minimize or avoid such defects, a precise understanding of the process is essential. Simulative approaches allow for a basic design of the mold filling process, but due to many influencing variables, the real behavior cannot be exactly reproduced. For this reason, there are already various approaches to process monitoring in RTM and HP-RTM using different sensors.

In [3], one of the first approaches is presented, which used frequency-dependent electromagnetic sensing (FDMES) to monitor the viscosity and degree of cure of the resin in RTM molds. For this purpose, the time, temperature, and amplitude of the frequency-dependent complex dielectric constant were correlated with the degree of cure and viscosity of a given resin. The mathematical relationships derived from this were used to predict the physical state during an actual run. Further studies [4,5,6] also show the successful use of dielectric sensors for the characterization of the resin system and visualization of the flow front in the RTM process as well as the associated process optimization. Using an area sensor array composed of electrodes, researchers succeeded in obtaining the effects of the process parameters on the mold filling behavior of the vacuum-assisted resin transfer molding (VARTM) process by determining the electrical impedance [7,8]. The influence of the viscosity and temperature of the epoxy resin on the shape of the flow front was investigated. Furthermore, pressure sensors were used in [9,10] to analyze the RTM process. This approach also allowed for the permeability of the fiber semi-finished product to be determined experimentally in order to validate the existing simulative approaches.

The transferability of the approaches mentioned above for monitoring the HP-RTM process on an industrial scale is essentially limited by two factors. Firstly, in the case of internal sensors such as pressure sensors or electrode arrays presented among others in [11], they must already be taken into account in the design phase of the mold. On the other hand, these internal sensors leave marks on the finished part, which may be undesirable, especially for FRP parts that are used in the field of view. Various approaches describing the use of ultrasonic sensors offer the advantage that they can also be attached to the outside of the mold. Ultrasonic sensors are also used for monitoring the curing process in RTM molds by studying the influence of the degree of curing on the sound velocity of a transmitting sound wave [12]. The authors in [13] were also able to draw conclusions about the three-dimensional flow behavior of the resin by evaluating the transit time of two ultrasonic sensors placed on both sides of the cavity. In addition, using planar piezoelectric elements in a combined reflection/transmission setup to draw conclusions about the flow velocity and shape of the flow front on the basis of the amplitude gradient during the mold filling process was successful in [14].

However, when transferring these approaches to the HP-RTM process, the problem arises is that the molds have to be designed to be much more sturdy due to the high process pressures; thus, a steel shell several centimeters thick has to be penetrated in order to obtain information from inside the cavity. It can be seen in [15] that due to the directional characteristic and resulting near-field length of the sensors used, the quality of the results decreases with increasing mold thickness. Furthermore, the increasing attenuation leads to a rather low signal-to-noise ratio (SNR). Therefore, a method is needed that can be used on the outside of the mold despite large mold thicknesses and that can also provide information about the flow front and its flow velocity with good spatial resolution.

This work deals with the use of linear phased array ultrasound for in situ monitoring of the flow front. This can be used as a parameter for process stability in the HP-RTM process and therefore for simple process monitoring [16,17]. For this purpose, various process parameters are determined during the production of a demonstrator component and compared with the results of a mold filling simulation. In this way, it is also possible to optimize the mold filling simulation with regard to local differences in the compaction of the fiber semi-finished product. In contrast to the previously mentioned studies, the use of linear phased array technology means that an established non-destructive testing instrument can be used, as is also the case for conventional standardized testing. This means that almost no adjustments are required, and the effort to integrate the instrument into the production process is minimized.

## 2. Materials and Methods

### 2.1. Ultrasound Measurement Principles

**Figure 1:**
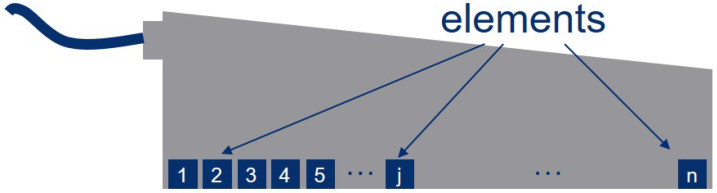
Schematic structure of a PAUT probe.

Phased array ultrasonic testing (PAUT) describes the use of ultrasonic probes, which are usually made up of 8 to 64 individual elements (Figure 1) [18,19]. Phase-controlled excitation of the individual elements enables electronic manipulation of the sound field in the form of angular scanning, focusing, or sequential perpendicular scanning.

The experimental procedure with a PAUT probe is based on the principle that the arrival of the resin front on the elements can be determined by the reflection coefficient *R* of the interface between the mold and the cavity. For *R*:

(1) $$R=\frac{Z_{2}-Z_{1}}{Z_{2}+Z_{1}}\;$$

where the acoustic impedance $Z_{1}$ and $Z_{2}$ is given by

(2) Z=ρ·c,

where ρ is the density and *c* is the sound velocity. Thereby, a value of *R* of 1 or −1 corresponds to a total reflection and a value of 0 corresponds to a complete transmission of the sound energy at the interface.

In addition to the experimental approach, a numerical approach according to Formula (1) is used to quantify the reflection coefficient. It is necessary to know the sound velocity of the resin, and therefore, the reflection coefficient is dependent on the reaction time. Therefore, the acoustic properties of the resin system used in these experiments are determined by an experimental test setup. The sound velocity is measured under near-process conditions as a function of the reaction time.

By sending and detecting with each element of the probe individually one after another, the PAUT technology enables the detection of the spontaneous switch of the acoustic properties. Due to the small element pitch of 0.85 mm, this results in a decisive advantage in spatial resolution and is therefore an extension of the approaches already presented.

In PAUT, the individual elements can be assumed to be point sources due to the low element size to wavelength ratio. It is therefore possible to generate a plane wave front by exciting a group of individual elements (aperture) following the Huygens principle [20]. The interaction of this plane wave front with the investigated interface and therefore the detection of the reflected signal is primarily in phase. This leads to a higher amplitude and therefore a better SNR. Moreover, by using the phase-shifting abilities of the PAUT technology, the elements in an aperture can be focused on a specific spot to enhance the beam focus in the distant far field [21].

### 2.2. Materials

The materials to be penetrated by the ultrasonic waves are the delay line, the tool material, the resin, and the fibers. The delay line is composed of polymethylmethacrylat (PMMA) with a density of 1.18 g/cm³. The temperature-dependent sound velocity has a linear course, starting from 2759 m/s at 23 °C to 2581 m/s at 80 °C. The mold used in the HP-RTM process is made of a steel type 1.2312 tool (40CrMnMoS8-6) with a density of 7.85 g/cm³. The fiber-reinforced composite that is created consists of the polyurethane (PUR) resin Henkel Loctite Max 2 and the glass fibers Owens Corning SE 1200. The PUR resin system is a two-component system (mixing ratio 100:128) with an internal release agent mixed in. This results in a calculated density of 1.13 g/cm³. The glass fibers are in the form of a single end roving, which is best suited for use in braiding. The glass roving has a yarn fineness of 2400 tex and has approx. 4500 filaments, with each filament having a diameter of 16 µm. Glass fibers themselves have a density of approx. 2.6 g/cm³.

### 2.3. Part Geometry and Manufacturing Process

**Figure 2:**
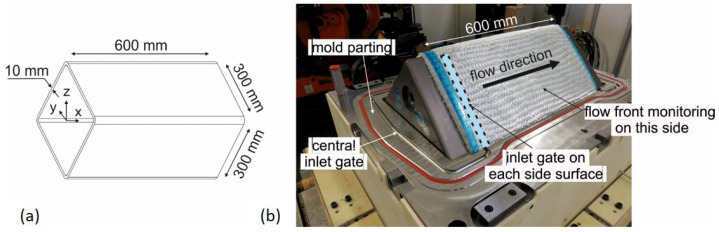
Geometry of thick-walled glass fiber-reinforced composite structure (**a**) and mold parting plane with preform on mandrel placed in HP-RTM measuring tool before injection (**b**).

Resin and fibers are consolidated in a high-pressure resin transfer molding process (HP-RTM) into a 10 mm thick-walled rectangular fiber composite hollow structure. The dimensions are shown in Figure 2. The manufacturing process is a two-stage process. In the first step, the dry glass fiber preform is created by braiding 288 rovings in 16 layers with an orientation of ±45° onto a mandrel. In the second step, the fibers are impregnated with resin, and the laminate is cured in the high-pressure resin transfer molding process.

The 16-layer braid, created by guiding a mandrel with a robot back and forth through the center of the braiding wheel (Herzog RF 1/288-100), does not have a homogeneous fiber architecture. This is due to the rectangular shape of the mandrel compaction of the fibers being higher in the edge areas compared with the flat sides of the mandrel. Furthermore, a deviation of the desired fiber orientation occurs on the flat sides of a squared mandrel when compared with braiding on a cylinder [22,23,24,25]. In addition, each change in braiding direction causes a run-in zone where the fibers need to align themselves to a constant pattern. These effects lead to local deviations in fiber orientation, non-constant spacing between rovings, and locally varying compaction, which in turn affect the resin impregnation and local mechanical properties [26] of the cured part.

**Figure 3:**
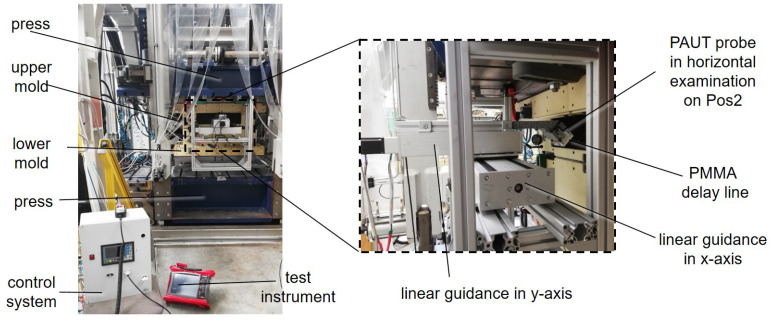
HP-RTM complex with scan unit for flow front monitoring.

After braiding, the mandrel with the braid is transferred to and placed in the HP-RTM measuring tool. It is a two-part mold for use in a shuttle mold carrier (Figure 2b). The parting plane is off center by 20 mm and runs through two opposite corners of the component to prevent fiber jamming in the parting plane when the mold is closed. Due to process-related deviations (run-in area) and variations in yarn count, the preform weight varies in the range of 9043 ± 157 g. The mandrel with the fibers is weighed to determine the preform mass to calculate the amount of resin required for each resin injection to achieve the maximum possible internal mold pressure of 50 bar (limited by the clamping force of the shuttle mold carrier) for pore-free consolidation. Before resin injection, the HP-RTM measuring tool is heated to 80 °C and evacuated to a residual pressure <2 mbar. Resin injection is carried out with a high-pressure metering machine (FRIMO PUReMix) at a mass flow rate of 30 g/s at an approx. resin temperature of 45 ± 1.5 °C. The mean value of the discharged resin quantity is 4454 ± 77 g with a filling time of 149 ± 2 s depending on the preform weight. The curing time is 30 min at 80 °C. Resin injection takes place via the line gate (see Figure 2b). To monitor the flow front with the PAUT probe at variable positions, a pocket was designed into the HP-RTM measuring tool (Figure 3). The wall thickness in this area accounts for up to 60 mm to prevent mold deformation at 50 bar internal pressure.

### 2.4. Process Simulation—Mold Filling

**Figure 4:**
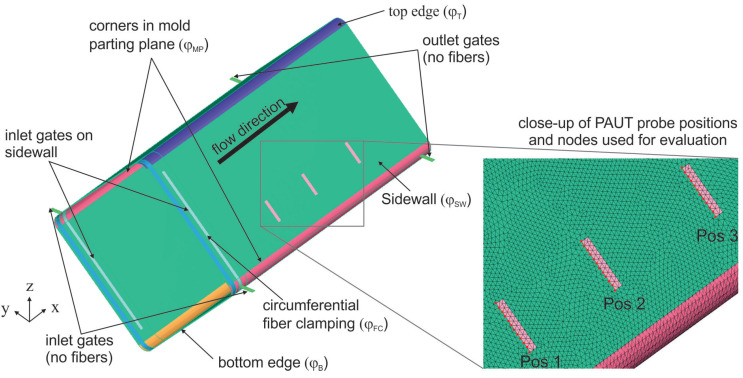
PAM-RTM simulation model for mold filling simulation with different zones of element properties.

In the design phase of the mold and process, a mold filling simulation using ESI PAM-RTM software was carried out to determine a gate geometry for a flow front through the fiber preform that progresses as evenly as possible. The goal was to avoid inlet gates on the later part area and meeting flow fronts. Therefore, a flow front direction along the axis of the part with a constant mass flow rate of 30 g/s was chosen. A shell model with tria elements with edge lengths of 5 mm with a total number of 63904 elements was built. A constant viscosity of 200 mPas was used for all simulations, and gravity was considered. Fluid inlets and outlets were placed on the edges of the shell elements. Fiber volume content (φ) and in-plane permeability $K_{1}$ and $K_{2}$ and out-of-plane permeability $K_{3}$ as well as thickness were adjusted for different areas of the model, respectively. Gate areas, fiber clamping areas, and areas of undisturbed fiber were considered (Figure 4). Fiber clamping areas were taken into account by employing $\varphi_{FC}=0.8$ as well as reducing the permeability by a power of ten. The element properties of shell elements used for mold filling simulation can be found in Table 1.

Figure 2 and Figure 4 also shows the gate variant implemented in the high-pressure RTM mold. It consists of two gates in the mold parting plane, each of which extends into two line gates on the adjacent sidewalls. The line gates are not circumferential to avoid resin runners in the upper and lower corner of the structure. In the experimental PAUT measurements, an uneven flow front was detected. Therefore, the model for the mold filling simulation is refined to allow for a comparison with the ultrasonic data obtained from the experiments. In addition to refining the mesh (82,642 elements), nodes are inserted at the exact positions of the PAUT probe. An area is defined where the left outside edge represents the mid line of the PAUT probe at each position. The distance between each node represents five times the PAUT element size. The arrival of the flow front is evaluated by taking the time when the filling factor for the node switched to one. Furthermore, zones of different φ are implemented into the model to simulate different compaction scenarios. Thus, there is a zone for the sidewalls named $\left(\varphi_{SW}\right)$, the edges of the preform due to braiding and closing of the mold $\left(\varphi_{MP}\right)$, and the top as well as bottom $\left(\varphi_{TB}\right)$ of the profile. These zones can be assigned higher or lower fiber volume content permeabilities, respectively, in the simulation model.

Process studies can be carried out in order to analyze the influence of compaction scenarios on the flow front position and velocity. For the validation, the PAUT measurement can be used.

## 3. Acoustic Characterization and Simulation

**Figure 5:**
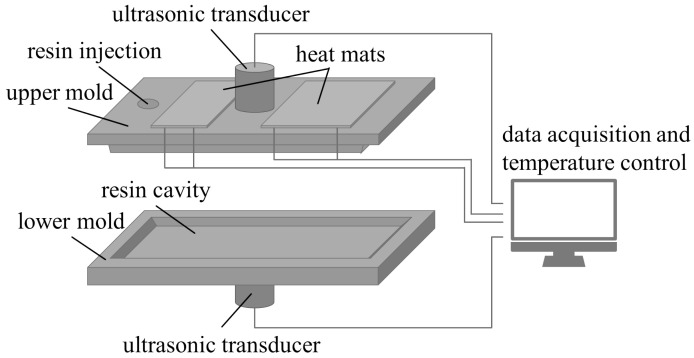
Experimental setup for the resin characterization.

In order to perform a valid evaluation and interpretation of the ultrasound data, a rough estimation of the expected results is necessary. For this reason, *R* was calculated by determining the sound velocity of the resin used in the following experiments. Therefore, a testing mold was constructed with which an ultrasonic transmission test can be carried out under near process conditions (Figure 5). To achieve these conditions, the test setup can be heated to process temperatures using two heating mats with integrated thermoelements that ensure the temperature control. In this case, the mold was preheated to 80 °C. Because the process pressures of a real HP-RTM process could not be realized with the experimental setup, this influence had to be neglected for the characterization.

**Figure 6:**
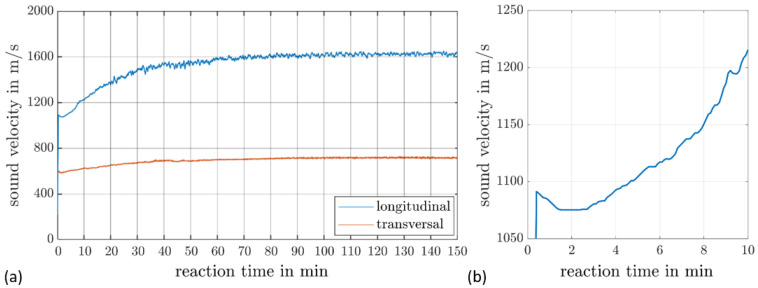
Longitudinal and transversal sound velocity of the resin system as a function of the reaction time (**a**); a detailed view of the longitudinal sound velocity at the start of the reaction (**b**).

For the experiments, the resin was preheated to 50 °C and manually poured into the mold. From this point, ultrasonic transmission measurements were started and performed over a period of 9000 s, during which the longitudinal and transverse sound velocities were determined at 1 MHz. The results of the preliminary test are shown in Figure 6a.

Comparable to references elsewhere such as [27] or [28], the course of the sound velocity follows a roughly sigmoidal curve. In addition to this consistency, the following two deviations were noted. On the one hand, a very slight decrease in the sound velocity at the beginning of the course can be seen in Figure 6b. At this point, the resin is still in a liquid state and has not yet developed a viscoelastic character. Accordingly, an almost constant sound velocity can be expected. The temperature difference between the resin and the mold, however, causes the resin system to heat up, which explains the drop in the sound velocity [29]. The second deviation is that the transverse wave was already detected at the start of the curing process. In contrast to the experiments of [27], a test frequency that was a factor of 10 lower was selected for the experiments carried out. As a result, the attenuation, an effect that increases with an increasing frequency, was significantly reduced. Furthermore, the fast-reacting resin system seems to allow for an earlier evaluation of the shear wave velocity.

An important finding for further investigations and for the numerical determination of the reflection coefficient is that in the first 300 s of the curing process, an almost constant sound velocity is present. Although the temperature influence is measurable in this range, it only leads to a deviation of approx. 1%, which is a negligible influence for the later evaluation.

Based on the resin characterization, Formula (1) and (2) can be used to calculate the reflection coefficient R and thus the expected amplitude drop at the interface between the HP-RTM mold and the cavity inside of it. For the calculation, the influence of the fiber was neglected as it has just a minor effect. Before infiltration, R≈−1 is valid for process-related reasons, as a vacuum prevails in the cavity. The density of the mold is given in Section 2.2, where the sound velocity and parameters for the PUR-resin were determined experimentally at process temperatures. The density of the PUR corresponds to those of the unreacted individual components as the results of the resin characterization suggest a measurable start of the reaction only from about 300 s and thus after complete mold filling. The influences caused by the temperature differences between mold and resin were not taken into account. The values and result of the calculation can be found in Table 2.

It follows from the calculation that the proportion of reflected sound energy from the unfilled mold is approx. 8% higher than at the interface with the resin-filled mold. Transferred to the ultrasonic measurement, the amplitude of the echo of this interface should decrease by the same amount after the arrival of the resin.

When evaluating ultrasonic data, the signal-to-noise ratio is an important parameter that provides an indication of the quality of the data obtained. The higher this value, the better the data can be analyzed and the higher the information content that can be derived from the evaluation. It is therefore necessary to optimize the settings for a PAUT measurement for this very purpose. For the following experiments, the average of a measurement χ over *N* repetitions was calculated with an average function $\bar{\chi {\left(k\right)}_{n}}$ for *k* sample points and *n* single elements of the PAUT probe [30] in order to achieve a higher SNR:

(3) $$\bar{\chi {\left(k\right)}_{n}}=\frac{1}{N}\sum_{j=1}^{N}\chi_{j}\left(k\right).$$

To achieve a further improvement in the SNR, the measurements can be performed with an aperture consisting of a specific number of individual elements of the PAUT probe. In order to choose a suitable aperture, it must be considered that it must be high enough to achieve a sufficient amplitude height at the inspection depth of 60 mm. Furthermore, due to the combination of individual elements, the achievable spatial resolution and the area in which the flow front can be detected decreases with an increasing aperture.

To select an aperture for the experiments, the amplitude at the depth of the later evaluation was simulated with different apertures. The simulation was carried out using CIVA NDE (EXTENDE, Massy, France) and the specifications in Table 3. In addition, a phase-delayed excitation of the elements was used for the 8 and 12 elements apertures to achieve a focusing of the signal at the investigated interface between the HP-RTM mold and the cavity underneath the center of the probe.

**Figure 7:**
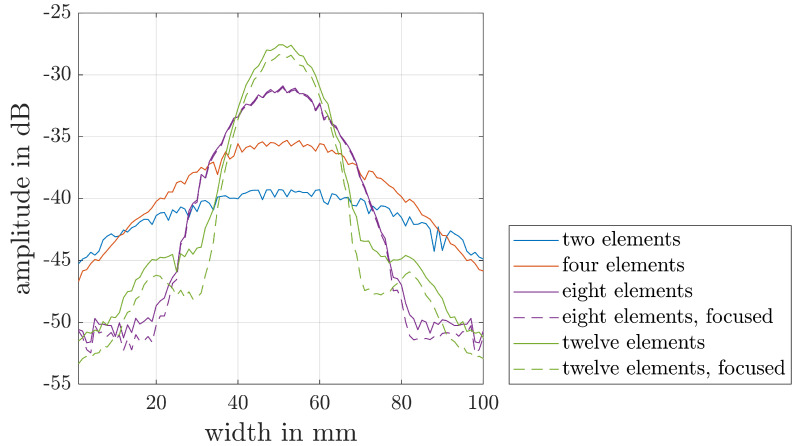
Simulated amplitude of the ultrasonic signal at the interface between the HP-RTM mold and the cavity.

The results of the simulations are shown in Figure 7. It can be seen that as the number of elements combined in an aperture increases, the focusing of the signal increases as well. Similarly, the focusing produced by the time-delayed excitation shows no significant effect on the achievable signal amplitude for the simulated apertures. On the basis of these investigations, an unfocused aperture of eight elements was chosen for the experimental studies. Taking into account the specifications of the PAUT probe that was used, a spatial resolution of 0.85 mm over a length of 48.45 mm was achievable for the following experiments. Due to the aperture, an averaging of the measured values similar to a moving average must be taken into account. The aperture and element pitch therefore result in an averaging over a window of 6.8 mm.

## 4. Experimental Flow Front Monitoring

The ultrasonic examinations were performed using a phased array probe with 64 elements, a single-element pitch of 0.85 mm, an element width of 10 mm, an active length of the probe of 54.3 mm, and a frequency of 2 MHz. A 20 mm PMMA delay line was used to ensure thermal decoupling of the temperature-sensitive probe. The ultrasonic measurements were performed over a period of 200 s with ten measurements per second and a signal amplification of 55 dB. To improve the signal-to-noise ratio, each individual measurement was averaged 32 times. Following the results are in Section 2.4, where the measurements were performed with an unfocused aperture of eight elements.

**Figure 8:**
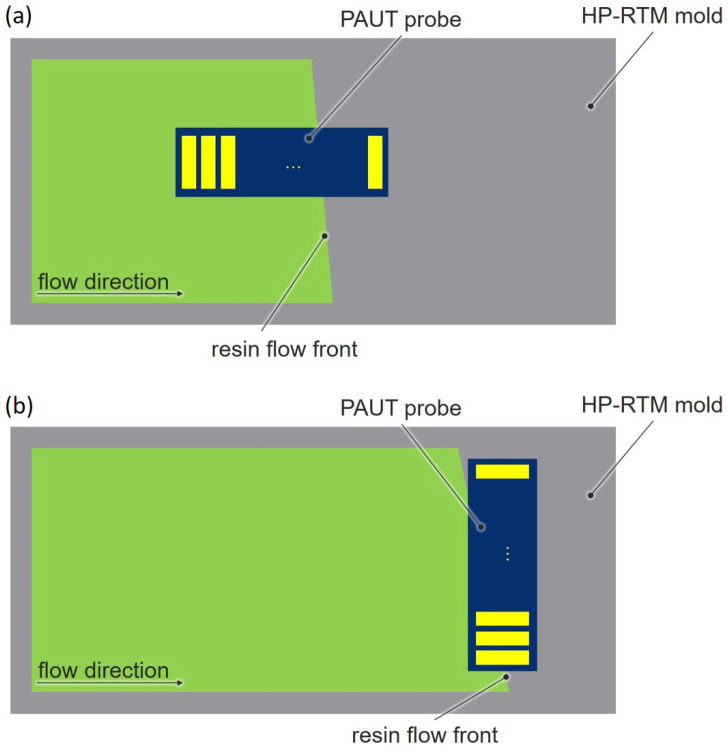
Measuring principle of flow front detection. Each element (yellow) detects the flow front individually. Setup (**a**) is used to determine the flow velocity and setup (**b**) is used to determine the shape of the flow front.

To obtain the maximum amount of information, the sensor is mounted in two orientations. The velocity of the flow front is determined with an horizontal sensor orientation according to v=s/t with the different detection times of the individual elements *t* and the geometry of the probe *s*. The shape of the flow front is recorded with a vertical sensor orientation by considering the detection time of each element and the velocity measured in the horizontal configuration (Figure 8).

**Figure 9:**
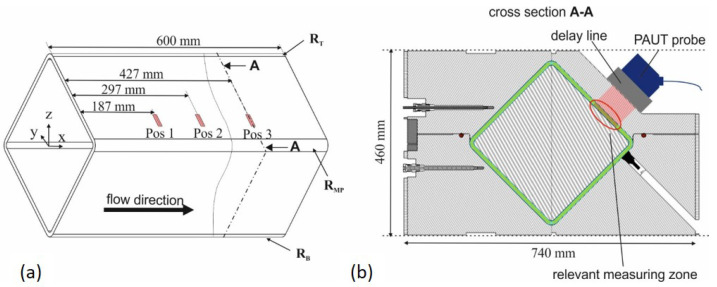
Depiction of the three measuring positions on the square test structure examined during resin injection (**a**) and sectional view of the HP-RTM mold with the cavity (green) and the measuring area of the PAUT probe (**b**).

For the PAUT measurements, the HP-RTM mold was accessible from the side (Figure 3 and Figure 9). The thickness of the mold at the measuring point was 60 mm. In addition, a 20 mm thick PMMA delay line was used for the experiments, which provided a thermal protection of the PAUT probe from the mold. The probe was then placed on the mold in the three test positions already described.

In order to determine the parameters during the mold filling process, three measuring positions were defined along the test structure. These are located along the flow direction at 187 mm (Pos1), 297 mm (Pos2), and 427 mm (Pos3) intervals. Ultrasonic examinations, one horizontal and one vertical, were carried out at each of the measuring positions (Figure 9). Due to the size of the sensor and the limited space available, the sensor position could be varied in the X direction only.

## 5. Results

### 5.1. Process Studies

With the refined simulation model, process studies were carried out. The first process study was carried out with a constant permeability φ for the side and edge areas of the preform and represents the initial simulation. In the process studies, the local compaction at the edges of the preform due to the braiding and closing of the mold was varied; meanwhile, the fiber volume content in the fiber clamping $\varphi_{FC}=0.8$ and the sidewalls $\varphi_{SW}=0.5$ was kept constant in all simulations.

**Figure 10:**
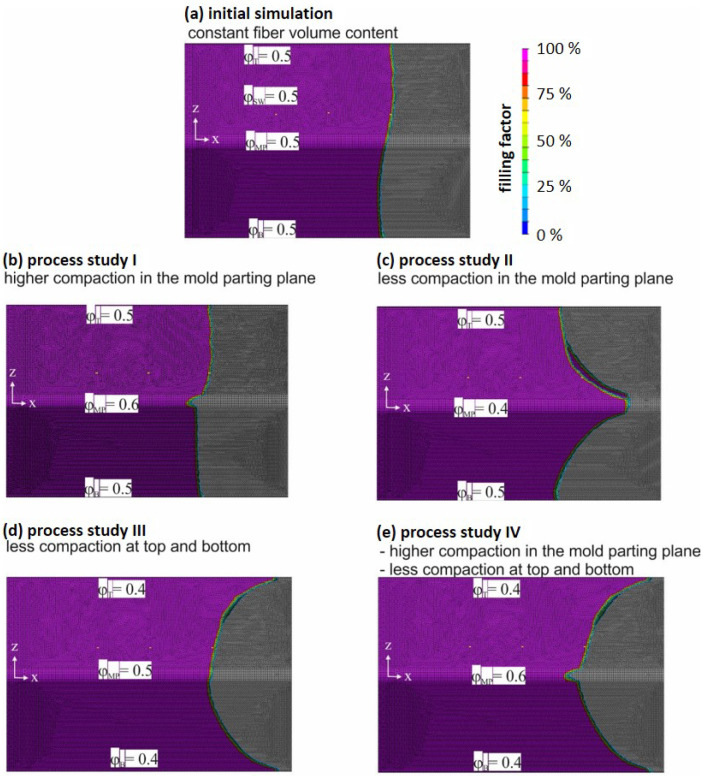
Simulation result of flow front progression at 75% mold fill for different compaction scenarios of the glass fiber braid in the mold parting plane and at top as well as bottom edges of the preform.

Settings of φ for the preform edges in the mold parting plane $\left(\varphi_{MP}\right)$ and at the top $\left(\varphi_{T}\right)$ as well as bottom $\left(\varphi_{B}\right)$ of the profile and simulation results can be seen in Figure 10 (one can find corresponding permeabilities in Table 1).

The simulation results show that increasing the fiber compaction impedes the resin flow, whereas less compaction enhances the flow. The effects on the shape of the flow front can be seen in Figure 10. It is found that higher compaction in the mold parting plane does not change the overall shape of the flow front in the flat surface of the preform. In contrast, less compaction in the mold parting plane alters the shape of the flow front as this resembles a resin runner Figure 7d. The latter is unlikely to occur in practice. In the experiments, it was observed that fibers will be pushed towards and compressed in the mold parting plane when the mold is closed. This circumstance was already taken into account in the design phase of the mold by an asymmetric location of the mold parting plane.

**Figure 11:**
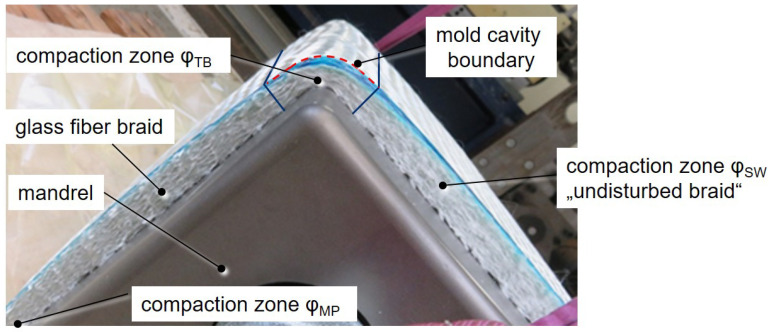
Compaction zones of glass fiber braid at the corners of the mandrel.

At the radii of the top and bottom edges, high preform compaction is observed during the preform process (Figure 11). If this greater compaction is mapped with a higher fiber volume content in the shell elements, an unrealistic simulation result is obtained because the shell elements assume homogeneous properties in the thickness direction. However, the compaction in the edge area is much higher than in the side surfaces of the preform, so that the preform thickness in the edge region is already lower than the cavity height (dashed line in Figure 11). Therefore, in reality, a flow channel for the resin exists. In approximation, this effect can only be represented with a locally lower fiber volume content (or higher permeability) for the shell elements at the top and bottom edges. Figure 10c,e show that less compaction (flow channel) at the top or bottom edge of the preform results in a higher flow in these areas, which alters the shape of the flow in the flat surfaces significantly. The progression of the flow front is faster towards the top and bottom of the profile.

### 5.2. PAUT Measurement under HP-RTM Conditions

**Figure 12:**
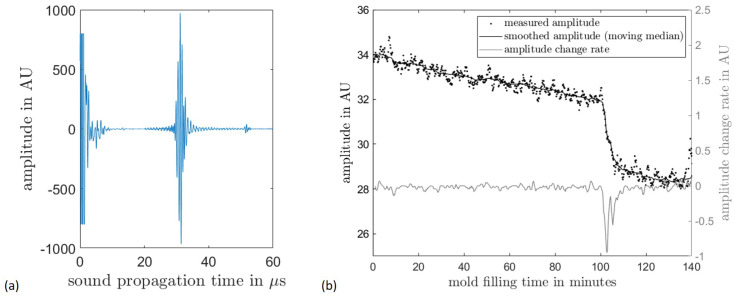
Amplitude course of a PAUT element over the sound propagation time (**a**) and over the mold filling time (**b**).

To evaluate the experiments performed in the HP-RTM process, the sound propagation time of each PAUT element was examined. In the amplitude scans obtained in this process (Figure 12a), a distinction can be made between three echoes: the entrance echo, starting at 0 µs; the back wall echo of the PMMA delay line, starting at approx. 28 µs; and the echo of the interface between mold and cavity at approx. 50 µs. As mentioned above, the amplitude of this third echo was used for further evaluation.

For the evaluation, the course of said amplitude was determined for each PAUT element as a function of the mold filling time (Figure 12b). The A-scan appears to be rather noisy, which is due to the signal losses in the interfaces located in the measuring range. Therefore, a high signal amplification was necessary, which significantly amplifies the existing thermal noise. In order to reduce this noise, a smoothing moving median filter was applied with a window of 50 measured values, which corresponds to a time period of 5 s. Based on the smoothed curve, the rate of change of the amplitude was calculated over five measurement steps or 0.5 s.

Two phenomena can be observed when evaluating the amplitude curve. First, a steady decrease in amplitude due to the temperature rise and the associated increase in acoustic damping in the delay line can be observed. In addition, a sharp drop in amplitude of about 9% is observed at about 106 s, which is due to a jump in the reflectivity of the investigated interface caused by the flow front. Accordingly, the measured change of the reflected amplitude is very close to the calculated value of about 8%. The time of the amplitude drop is determined for each element and is evaluated as the arrival of the flow front. For this purpose, the time of the minimum of the rate of change is determined. The position of the elements in the probe as well as the global position of the probe thus allow for the reconstruction of the flow velocity for a horizontally oriented measurement. In the case of a vertically aligned measurement, the time of arrival of the flow front can be registered. By including the flow velocity determined in the horizontal test, the shape of the flow front can thus be reconstructed.

These results are at first compared with the data determined from the initial simulation with homogenous compaction of the glass fiber braid (constant permeability φ for the side and edge areas of the preform). For this purpose, the detection time at which the flow front could be detected at the respective measuring points and the flow rate are compared. The results are shown in Table 4.

When comparing the detection time of the flow front, a significant deviation between the initial simulation with homogeneous compaction and the measurement can already be observed. At measurement position one (Pos1), a delay of the measured flow front of 3.3 s has been recorded compared with the simulation. Over the course of the flow front, an increasing lag of the measured detection time of up to 8.4 s at measurement position three (Pos3) can be seen.

While the flow velocity in the initial simulation remains largely constant with a slight decrease at position three, a significant and almost linear increase can be observed in the measured values. Especially at the beginning, the flow velocity at Pos1 is overestimated in the simulation, which also explains the deviating detection times of the flow front.

**Figure 13:**
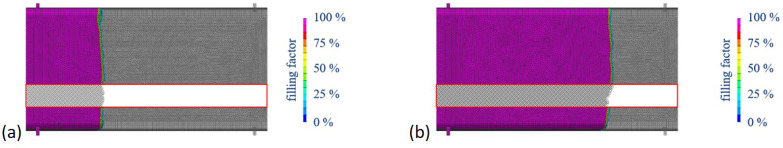
Shape of the flow front derived from the initial mold filling simulation and the PAUT measurements (red marked) at measuring positions Pos1 (**a**) and Pos3 (**b**).

Another characteristic that can be considered is the shape of the flow front. For the evaluation of the shape of the flow front, the graphical simulation results are considered (Figure 13), in which the flow front (purple) can be seen arriving from the left. In addition, the flow front determined from the ultrasonic data (hatched area in the red marked section) is superimposed on the plot for comparison. It should be noted that the temporal differences between measurement and simulation are not taken into account in this view. While there is a clear deviation between simulation and measurement in both the temporal and flow velocity observations, no significant difference can be seen in a purely visual comparison of the shape of the flow front. Such an observation is therefore only helpful for a rough preliminary estimation due to the limited observation range.

### 5.3. Validation of the Process Studies

Since the PAUT data are also used as the basis for optimizing the mold filling simulation performed, a quantifiable value must be introduced for the comparison between simulation and measurements. For this purpose, the root mean square error (RMSE) between the observed data of the PAUT measurements *O* and the expected data from the simulation *E* over all measuring points *n* is calculated using Formula (4). The smaller the value, the better the experiments fit the simulation.

(4) $$RMSE=\sqrt{\frac{\sum {\left(O_{i}-E_{i}\right)}^{2}}{n}}$$

**Figure 14:**
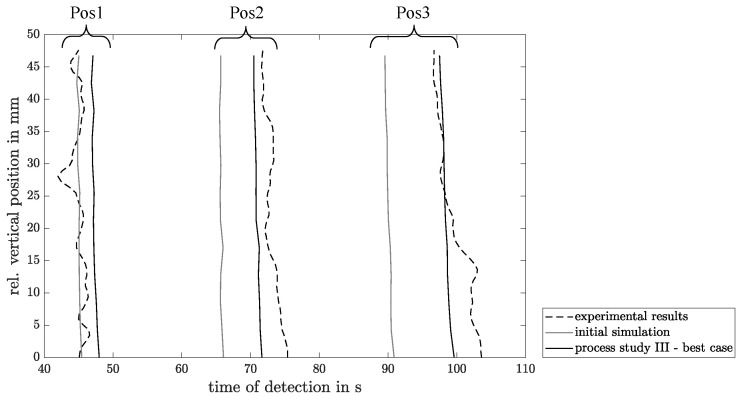
Visualization of the detection time of the measured and simulated flow front at Pos1–3.

The assumption of varying compaction in the radii of the test structure can be confirmed on the basis of the performed optimization of the simulation. Table 5 shows the calculated RMSE between the simulations performed and the measurements. As mentioned in Section 2.3, an increased compaction of the fibers can be assumed in the mold parting plane ($\varphi_{MP}=0.6$). Furthermore, in the upper and lower radii of the test structure, a lower compaction can be assumed ($\varphi_{MP}=0.4$), which supports the theory of the presence of a flow channel in this region. The results show that a decrease in the compaction in the radius of the top and bottom edges leads to a decrease in the RMSE by about 70% over the entire flow path of the test structure and thus to a better conformity with the measured values. While the adjustment of $\varphi_{TB}$ leads to a significant improvement, the adjustment of the compaction $\varphi_{MP}$ does not significantly change the RMSE and thus has a minor effect on the mold filling process. In relation to the mean RSME, the following can be seen that the process study III with the settings of $\varphi_{MP}=0.5$ and $\varphi_{TB}=0.4$ shows, with an average of 2.25 s, the best case for the simulations carried out. This is also shown graphically in Figure 14 by comparing the detection time of the initial simulation, the best case simulation, and the PAUT-measured values, respectively.

## 6. Conclusions and Outlook

It was shown that the PAUT measurement method presented here can be used to determine the detection time, velocity, and shape of a flow front in an HP-RTM process with a thick-walled mold. By comparing the results with those of a mold filling simulation, it was also proven that this method can be used both for optimizing simulations and for process monitoring. A particular advantage results from the high spatial resolution of the results, which is linked to the pitch of the phased array probe and the aperture used. Thus, despite a locally limited measuring range, conclusions can be drawn about the global flow behavior in a large mold such as the presence of areas of high fiber compaction or flow channels in the mold, which significantly affect mold filling behavior. In order to confirm these conclusions, in further investigations, the monitoring method used should be extended by additional sensors distributed on the mold, e.g., single transducer probes. This would allow for a more global experimental observation. Likewise, an evaluation of the fiber volume content at different positions of the manufactured test structures could provide information on the compaction present during manufacturing.

A limitation of the reliability of the determined results, especially with respect to the flow front shape, results from the stability of the considered mold filling process. As at least two mold filling processes must be accompanied to determine the shape of the flow front, inaccuracies would arise in a process with a varying mold filling sequence, leading to a false interpretation of the results.

The other limitations of the methodology presented here relate primarily to the analyzability of the ultrasonic signal. Here, the signal-to-noise ratio is a decisive value. With the decrease in this value, additional steps like increasing the aperture or performing smoothing functions become necessary, which lead to a decrease in the information content, e.g., the realizable spatial resolution. For this reason, further optimization of this method should place great importance on improving the signal-to-noise ratio, e.g., by investigating the use of measurement and evaluation algorithms such as the total focusing method. These also promise to improve the depth resolution, making a three-dimensional evaluation of the flow front across the component thickness conceivable. 

## Figures and Tables

**Table 1:** Element properties of shell element used for mold filling simulation.

Element Parameter	Unit	Gate Area without Fibers	Gate Area with Fibers	Undisturbed Braid	Fiber Clamping	Locally Higher φ	Locally Lower φ
φ *	-	0	0.5	0.5	0.8	0.6	0.4
$K_{1}$ and $K_{2}$	m²	$1.6\times 10^{-5}$	$1.6\times 10^{-5}$	$1.6\times 10^{-10}$ **	$1.6\times 10^{-11}$	$2.13\times 10^{-11}$ ***	$1.2\times 10^{-9}$ ***
$K_{3}$	m²	$1.6\times 10^{-5}$	$1.6\times 10^{-5}$	$8.32\times 10^{-12}$ **	$8.32\times 10^{-13}$	$1.17\times 10^{-12}$ ***	$6.62\times 10^{-11}$ ***
Fiber orientation	°	-	±45	±45	±45	±45	±45
Element thickness	mm	5	10	10	9	10	10

* Indication in absolute values in this article; ** taken from the literature; *** permeability of undisturbed areas are multiplied/divided by a factor of 7.5.

**Table 2:** Material properties for determining the reflection behavior at the interface between the mold and the cavity.

Parameter	Symbol	Unit	Steel	PUR-Resin
Density	ρ	kg/m${}^{3}$	7850	1124
Sound velocity	c	m/s	5907	1670
Acoustic impedance	Z	kg/m${}^{2}$s	46.37 × 10${}^{6}$	1.88 × 10${}^{6}$
Reflection coefficient (steel vs. PUR-Resin)	R	-	−0.922	

**Table 3:** Parameters for PAUT simulation for the optimization of the achievable amplitude in the inspection depth.

PAUT Simulation Parameters	Unit	Values
Aperture	-	2; 4; 8; 12
Element pitch	mm	0.85
Element width	mm	10
Gap between elements	mm	0.1
Frequency	MHz	2

**Table 4:** Simulated and measured detection times and flow velocities of the flow front.

Measuring Point	Unit	Pos1	Pos2	Pos3
Detection time initial simulation	s	43.5	66.4	89.8
Detection time PAUT measurement	s	46.8	74.3	98.2
Flow rate initial simulation	mm/s	4.66	4.67	4.56
Flow rate PAUT measurement	mm/s	3.81	5.02	6.45

**Table 5:** RMSE of measurement and simulation with different simulation parameters.

Evaluation Position for RMSE Analyse	Initial Simulation	Process Studies			
		**I**	**II**	**III**	**IV**
	$\varphi_{MP}=0.5$ $\varphi_{TB}=0.5$	$\varphi_{MP}=0.6$ $\varphi_{TB}=0.5$	$\varphi_{MP}=0.4$ $\varphi_{TB}=0.5$	$\varphi_{MP}=0.5$ $\varphi_{TB}=0.4$	$\varphi_{MP}=0.6$ $\varphi_{TB}=0.4$
Pos1	0.79 s	0.99	2.05 s	2.36 s	2.13 s
Pos2	7.49 s	8.16	7.37 s	2.32 s	2.32 s
Pos3	9.83 s	11.11	9.63 s	2.07 s	2.35 s
Average	6.04 s	6.75 s	6.35 s	2.25 s	2.27 s

## Data Availability

Data are available on request.
